# Potassium-Promoted Limestone for Preferential Direct Hydrogenation of Carbonates in Integrated CO_2_ Capture and Utilization

**DOI:** 10.1021/jacsau.3c00403

**Published:** 2023-11-09

**Authors:** Shuzhuang Sun, Zheng Chen, Yikai Xu, Yuanyuan Wang, Yingrui Zhang, Catherine Dejoie, Shaojun Xu, Xin Xu, Chunfei Wu

**Affiliations:** ¶ School of Chemical Engineering, Zhengzhou University, Zhengzhou, 450001, China; ‡ School of Chemistry and Chemical Engineering, Queen’s University Belfast, Belfast, BT7 1NN, U.K.; § Department of Chemistry, Fudan University, Shanghai, 200433, China; ∥ Key Laboratory for Advanced Materials and Feringa Nobel Prize Scientist Joint Research Center, Frontiers Science Center for Materiobiology and Dynamic Chemistry, School of Chemistry and Molecular Engineering, East China University of Science and Technology, 130 Meilong Road, Shanghai 200237, China; ⊥ European Synchrotron Radiation Facility, Grenoble, 38043, France; # Department of Chemical Engineering, University of Manchester, Manchester M13 9PL, U.K.; □ UK Catalysis Hub, Research Complex at Harwell, Didcot, OX11 0FA, U.K.

**Keywords:** carbon dioxide, hydrogenation, selectivity, integrated CO_2_ capture and utilization, transition-metals free catalysts, reverse water−gas shift reaction, dual functional material

## Abstract

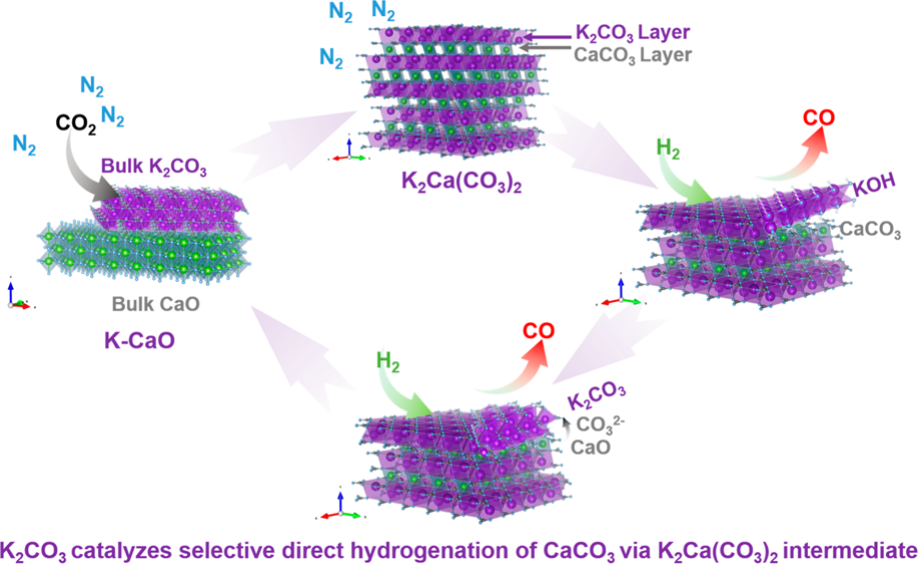

Integrated CO_2_ capture and utilization (ICCU) via the reverse water–gas shift (RWGS) reaction offers a particularly promising route for converting diluted CO_2_ into CO using renewable H_2_. Current ICCU-RWGS processes typically involve a gas–gas catalytic reaction whose efficiency is inherently limited by the Le Chatelier principle and side reactions. Here, we show a highly efficient ICCU process based on gas–solid carbonate hydrogenation using K promoted CaO (K-CaO) as a dual functional sorbent and catalyst. Importantly, this material allows ∼100% CO_2_ capture efficiency during carbonation and bypasses the thermodynamic limitations of conventional gas-phase catalytic processes in hydrogenation of ICCU, achieving >95% CO_2_-to-CO conversion with ∼100% selectivity. We showed that the excellent functionalities of the K-CaO materials arose from the formation of K_2_Ca(CO_3_)_2_ bicarbonates with septal K_2_CO_3_ and CaCO_3_ layers, which preferentially undergo a direct gas–solid phase carbonates hydrogenation leading to the formation of CO, K_2_CO_3_ CaO and H_2_O. This work highlights the immediate potential of K-CaO as a class of dual-functional material for highly efficient ICCU and provides a new rationale for designing functional materials that could benefit the real-life application of ICCU processes.

## 1 Introduction

**Figure 1:**
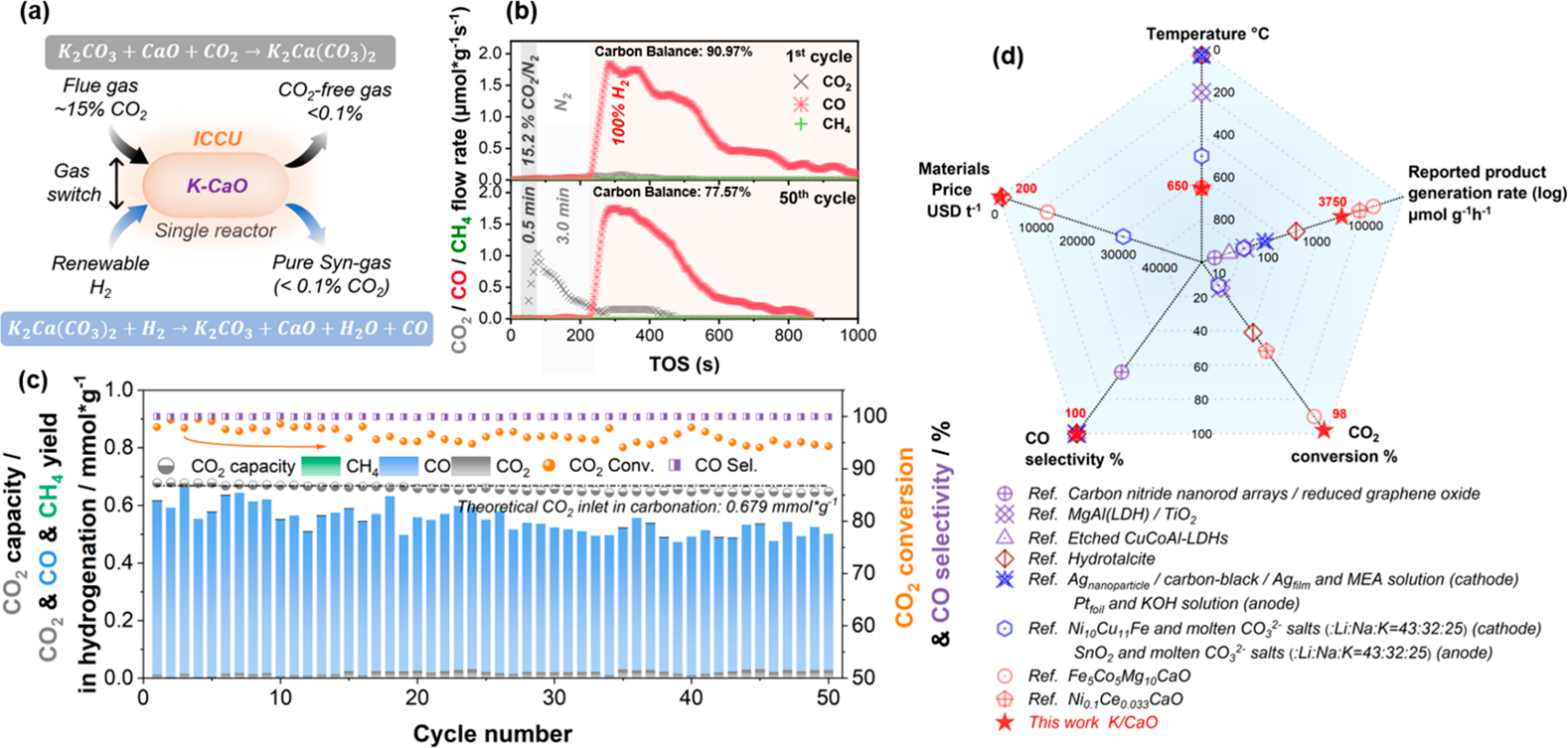
ICCU performances using 20 mol % K-CaO. ICCU schematic diagram and the pivotal reactions using K-promoted CaO (a). The real time (b) and cyclic (c) ICCU performance at 650 °C using 20 mol % K-CaO (carbonation: 15.2% CO_2_/N_2_ for 0.5 min; purge: N_2_ for 3.0 min; hydrogenation: 100% H_2_ to the end; carbon balance: C1 species during hydrogenation to CO_2_ capacities during carbonation). (d) Comparison of reported integrated CO_2_ capture and utilization systems.^9−12,14,16,25,26^

The emission of CO_2_ from using fossil fuels is a significant contributor to climate change.^1^ However, fossil fuels will still play a dominate role as a source of energy in the near future.^2^ Although the application of renewable energy (e.g., solar and wind energy) mitigates the challenges related to CO_2_ emissions, it faces shortcomings of high cost, low generation efficiency, and slow deployment.^3,4^ Consequently, upcycling CO_2_ to store renewable energy and keeping it in the carbon cycle provide practical strategies to address the above challenges. To shorten and simplify the whole CO_2_ capture and utilization processes, integrated CO_2_ capture and utilization (ICCU) processes have been proposed and studied.^5^ In a typical ICCU process, CO_2_ is first captured and fixed from diluted sources (e.g., flue gas) using sorbents, such as CaO and monoethanolamine.^6,7^ Subsequently, the saturated sorbents can be regenerated by converting the adsorbed CO_2_ into valuable chemicals, such as CO and CH_4._^8−10^ A wide range of catalytic processes have been attempted for the utilization process in ICCU, such as photocatalytic,^11,12^ electrocatalytic,^10,13^ plasma-catalytic,^14,15^ and thermo-catalytic^9,16,17^ processes (Figure 1d). The ICCU can occur under mild conditions (e.g., room temperature) with the assistance of photo- or electrocatalysts, representing a sustainable concept for reducing CO_2_ emission. However, they are limited by the low reaction efficiency (Figure 1d) and inhibited by the system complexity in scaling up.^18^ Compared to those processes, the conventional thermo-catalytic ICCU under relatively mild conditions presents a more practical solution to meet the urgent industrial level deployment requirement for carbon neutrality in the CO_2_ emission point, such as the power plants.^19^

Integrated CO_2_ capture and reverse water–gas shift (eq 1) reaction (ICCU-RWGS) is a promising route to reduce CO_2_ into CO using renewable H_2_. This combined with the Fischer–Tropsch process, liquid chemicals with long lifetime can be obtained from CO.^20^ It was widely believed that the CO_2_ is captured in the form of carbonates (eq 3) during CO_2_ capture step and then the gaseous CO_2_, decomposed from carbonates, react with H_2_ on the active sites of the catalysts in an integrated hydrogenation step.^9,16^ Hence, many researchers used the experience of catalyst design from conventional RWGS into ICCU-RWGS to enhance its performance.^8,9,16,17^ Typically, dual functional materials (DFMs), including active catalytic metals (e.g., Ni or Fe) and adsorbents (e.g., CaO), are widely applied in ICCU.^9,21,22^ However, due to the Le Chatelier principle-controlled equilibrium of RWGS and the occurrence of side reactions (e.g., eq 2), the performance of ICCU-RWGS is far from ideal. Furthermore, the introduction of active metals would increase the material cost and cause environmental distress.^23^
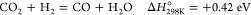
1
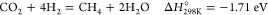
2
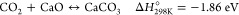
3Our previous study confirmed that CaCO_3_ could directly react with H_2_ to generate CO even in the absence of active transition metals (eq 4).^24^ However, the inevitable CaCO_3_ decomposition (reverse eq 3) restricted the CO_2_ conversion (<80%) at the regeneration/utilization stage. A significant amount of effort have been made to optimize RWGS catalytic performance, while there have been comparatively fewer studies that reveal the importance of the direct hydrogenation of carbonates. Here we propose a novel strategy to improve the selectivity of direct hydrogenation of carbonates (e.g., eq 4) by hindering the decomposition process (e.g., reverse eq 3), representing a more direct and effective route for CO_2_ utilization in ICCU.
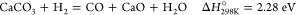
4Based on the above understanding, we report a transition-metal-free potassium-promoted CaO (K-CaO) DFM synthesized by a simple impregnation method. By introducing potassium into the commercial CaO, we find that the K species participate in the carbonation of CaO and form stable cocarbonates K_2_Ca(CO_3_)_2_ with enhanced reaction kinetics. The generated K_2_Ca(CO_3_)_2_ selectively reacts with H_2_ rather than decompose to generate CO_2_. As a result, we record a CO_2_ conversion rate >95% with ∼100% CO selectivity, which outperforms the results of ICCU processes carried out using state-of-the-art active metal (e.g., Ni)-based materials. Combining the experimental characterizations, such as ex-situ synchrotron radiation X-ray diffraction, Raman, and in situ diffused reflectance infrared Fourier transform spectroscopy analysis, with the density functional theory (DFT) calculations, we reveal the key intermediate and mechanism over the K-CaO DFM to facilitate the CO_2_ conversion and CO selectivity. Importantly, the K-CaO DFM presented here can be easily produced on a large scale at significantly low capital cost and harmlessly recycled as a cement additive after deactivation, as demonstrated in Figure 1a. This provides a circular solution for large-scale and cost-effective ICCU deployment with a zero-waste approach. Furthermore, this process can potentially be deployed by using two fluidized reactors for continuous CO_2_ capture and on-site utilization.

## 2 Results

The ICCU experiments were carried out in a single tubular fixed-bed reactor (Figure S1), and the carbonation and hydrogenation were realized by switching the inlet gas between simulated flue gas (15.2% CO_2_/N_2_) and H_2_. The K-CaO DFM was obtained by impregnating KNO_3_ onto CaCO_3_ and calcining at 800 °C (Figure S2). During the calcination, the KNO_3_ and CaCO_3_ were thoroughly decomposed and reformed into K_2_CO_3_, CaO, and K_2_Ca(CO_3_)_2_ (fairchildite), respectively (Figure S3 and S4). The commercial CaO reagent was applied as the benchmark material.

As demonstrated in Figure 1b, 20 mol % K-CaO can optimally achieve *ca*. 100% CO_2_ removal efficiency at 650 °C in 0.5 min of the carbonation process. Furthermore, the captured CO_2_ (carbonates) can be isothermally converted into CO with sustainable CO_2_ conversion (>95%) and CO selectivity (>99.9%) in 50 cycles of ICCU processes (Figure 1c and S5). The uncaptured CO_2_ in carbonation slightly increases from ∼0 to 0.008 mmol g^–1^ after 50 cycles, while the CO_2_ conversion only decreases to 94.3% in cyclic hydrogenation, which outperforms CaO (Figure S5d) and the reported ICCU-RWGS using transition-metal-CaO DFMs^8,9,16^ (Table S1) and other ICCUs using photo, electro or plasma-catalytic processes (Figure 1d). Even the molten salt slowly evaporates (Table S2) and the morphologies of materials slightly change in cyclic ICCU evaluations (Figure S13a). However, the main phase composition and surface species are stable (Figures S13b and c), consistent with outstanding performance stability. Notably, the trace transition-metal impurities (i.e., Fe, Pd, etc., in ppm level, reagents might contribute to the presented performance. However, an impact by Fe is unlikely given previous work^8^ showing that 10% Fe loading resulted in poorer performance (<85% CO_2_ conversion) compared to this work.

**Figure 2:**
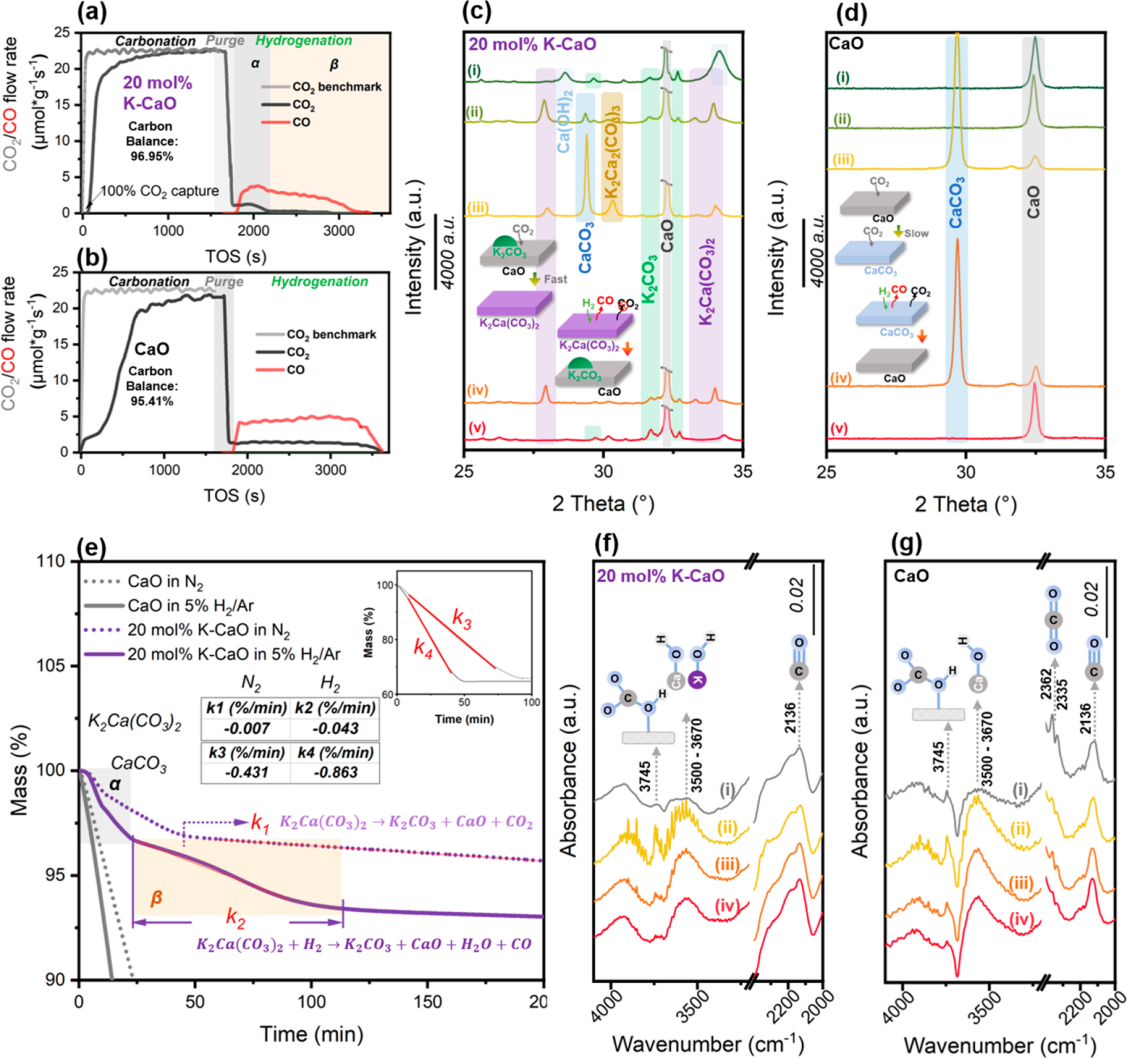
Mechanism investigation of CaO and 20 mol % K-CaO in ICCU. Real time ICCU performance using 20 mol % K-CaO (a) and CaO (b) at 650 °C (carbonation: 15.2% CO_2_/N_2_ for 30 min; purge: N_2_ for 3.0 min; hydrogenation: 100% H_2_ to the end; carbon balance: C1 species during hydrogenation to CO_2_ capacities during carbonation). (c) Ex-situ SRXRD patterns of 20 mol % K-CaO and (d) ex-situ XRD patterns of CaO in ICCU procedure (i: original material; ii: 0.5 min carbonation; iii: 30 min carbonation; iv: 10 min hydrogenation and v: end of hydrogenation; Note: the 2 Theta = 5° to 80° full patterns are shown in Figures S14 and S15). (e) The mass change of carbonated CaO and 20 mol % K-CaO in N_2_ or 5%H_2_/Ar at 650 °C (carbonation: 100 mL/min 15.2% CO_2_/N_2_ for 30 min) and in situ DRIFTs patterns of 20 mol % K-CaO (f) and CaO (g) in the hydrogenation step of ICCU at 650 °C (i: after 30 min carbonation and 10 min Ar purge; ii: 0.5 min hydrogenation; iii: 10 min hydrogenation and v: 60 min hydrogenation).

In order to demonstrate the superiority of short-term (0.5 min) carbonation and to understand the mechanism, we extended the carbonation time to 30 min. The ICCU performance using 20 mol % K-CaO is displayed in Figure 2a in comparison with the benchmark material of CaO (Figure 2b). Notably, there is an ∼1.7 μmol g^–1^s^–1^ CO_2_ escape flow using CaO in the initial carbonation stage at 650 °C, which does not occur using 20 mol % K-CaO. Furthermore, the saturated carbonated CaO and 20 mol % K-CaO display vastly different hydrogenation performances. More specifically, the amount of unconverted CO_2_ is constant and high (∼1.4 μmol g^–1^s^–1^) using CaO during the hydrogenation of ICCU at 650 °C (Figure 2b), resulting in a limited CO_2_ conversion (∼75%, Figure S6). In contrast, there are two hydrogenation stages for 20 mol % K-CaO. The α stage (time on stream, TOS, in the range of 1860–2180 s in Figure 2a) exhibits similar performance with CaO (Figure 2b), while the β stage (TOS of 2180–3300 s in Figure 2a) possesses a low CO_2_ flow (<0.2 μmol g^–1^s^–1^) and considerable CO flow (∼2.6 μmol g^–1^s^–1^), resulting in significantly enhanced CO_2_ conversion.

The influences of ICCU temperatures and K loadings were also investigated, as shown in Figures S6 and S7. It was found that the temperature was negatively correlated with the CO_2_ capture performance. Specifically, CaO could achieve excellent initial CO_2_ removal efficiency at 600 °C (Figure S7a, <0.6 μmol g^–1^ s^–1^ CO_2_ escape rate). However, the CO_2_ capture performance of CaO significantly deteriorates at higher temperatures due to the equilibrium of eq 3 (e.g., Figure S7c, >5.8 μmol g^–1^ s^–1^ CO_2_ escape rate). Notably, higher K loading can effectively improve the CO_2_ removal efficiency to 100% in the initial time period of carbonation, even at elevated temperatures. For example, 10 and 20 mol % K-CaO can completely capture CO_2_ in the simulated flue gas in the first ∼1 min of carbonation at 700 °C (Figure S7c). The high K loading (20 mol %) leads to a slight reduction in the catalyst porosity (Table S2) and CO_2_ capture capacity. However, it significantly promoted hydrogenation performance for ICCU. As demonstrated in Figures S8–S10, the unconverted CO_2_ released during the hydrogenation stage can be effectively prohibited with higher K loading, while the CO generation rate is only slightly affected. This indicates that the K element might participate in the formation of carbonates during the carbonation step and promote direct hydrogenation of carbonates by inhibiting the generation of CO_2_ in the decomposition step subsequent to hydrogenation. Furthermore, the stability of 20 mol % K-CaO is also verified in ICCU with 30 min carbonation condition (Figures S11 and S12).

To reveal the reaction intermediates, ex-situ synchrotron radiation X-ray diffraction (SR-XRD), ex-situ Raman, in situ diffused reflectance infrared Fourier transform spectroscopy (DRIFTs) and powder X-ray diffraction (XRD) were applied to demonstrate the changes to the crystal structure and surface groups of 20 mol % K-CaO at different stages of the ICCU process. The K-CaO samples were selected from five stages during ICCU at 650 °C for ex-situ characterizations, including (i) original as synthesized, (ii) after 0.5 min carbonation, (iii) after 30 min carbonation, (iv) after 10 min hydrogenation, and (v) in the end of hydrogenation, to monitor the evolution of the K-CaO material. The same experiments were also performed with CaO as the benchmark. For the original fresh sample, potassium and calcium mainly exist in the form of K_2_CO_3_, CaO, K_2_Ca(CO_3_)_2_^27,28^ (weak intensity), and Ca(OH)_2_ (Figures 2c and S14). After 0.5 min of carbonation, the signal intensity for K_2_Ca(CO_3_)_2_ significantly increases which is accompanied by the consumption of K_2_CO_3_ (eq 5), confirming the direct participation of K species in carbonates generation. There is a trace amount of CaCO_3_ generation on 20 mol % K-CaO in 0.5 min carbonation, consistent with Raman observation (Figure S15 and Table S3, weak CO_3_^2–^ bands at 711 and 1079 cm^–1^).^29^ As a benchmark, the CaO sample showed weak CaCO_3_ XRD peaks after 0.5 min of carbonation (Figures 2d and S16), indicating the slow kinetics of carbonation of CaO (eq 3). K_2_Ca(CO_3_)_2_ is identified as the key species for the 100% CO_2_ removal efficiency (Figure 2a and S7) during the carbonation using 20 mol % K-CaO. The as-formed K_2_Ca(CO_3_)_2_ might detach from the surface of CaO due to inappropriate crystal structure (Figure S17), and the continuous exposure of CaO contributes to the enhanced CO_2_ capture. The sufficient carbonation (30 min) then further promotes the formation of K_2_Ca(CO_3_)_2_ (Figures 2c, S14, and S15), accompanied by the enhanced generation of CaCO_3_. Furthermore, K_2_Ca(CO_3_)_2_ can partly form K_2_Ca_2_(CO_3_)_3_ by combing with CaCO_3_ (eq 6).
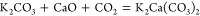
5
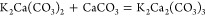
6After 10 min of hydrogenation (at the β stage in Figure 2a), the 20% K-CaO material presents mainly three crystal phases which can be attributed to K_2_Ca(CO_3_)_2_, K_2_CO_3_, and CaO (Figures 2c and S14). It is noted that the consumption of K_2_Ca_2_(CO_3_)_3_ and CaCO_3_ in the hydrogenation process is fast, which is responsible for the unreacted release of CO_2_ in the initial time of hydrogenation (α stage in Figure 2a). In contrast, K_2_Ca(CO_3_)_2_ tends to directly react with H_2_ to generate CO (eq 7) rather than self-decomposition to generate gaseous CO_2_ (eq 8), which is further evidenced by in situ DRIFTs (Figure 2f, no distinct CO_2_ generation). Importantly, by the end of the hydrogenation process, the CO_2_ saturated 20 mol % K-CaO catalyst is completely regenerated to the initial state (K_2_CO_3_ + CaO) (Figures 2c and S14) and ready for the next ICCU cycle.

**Figure 3:**
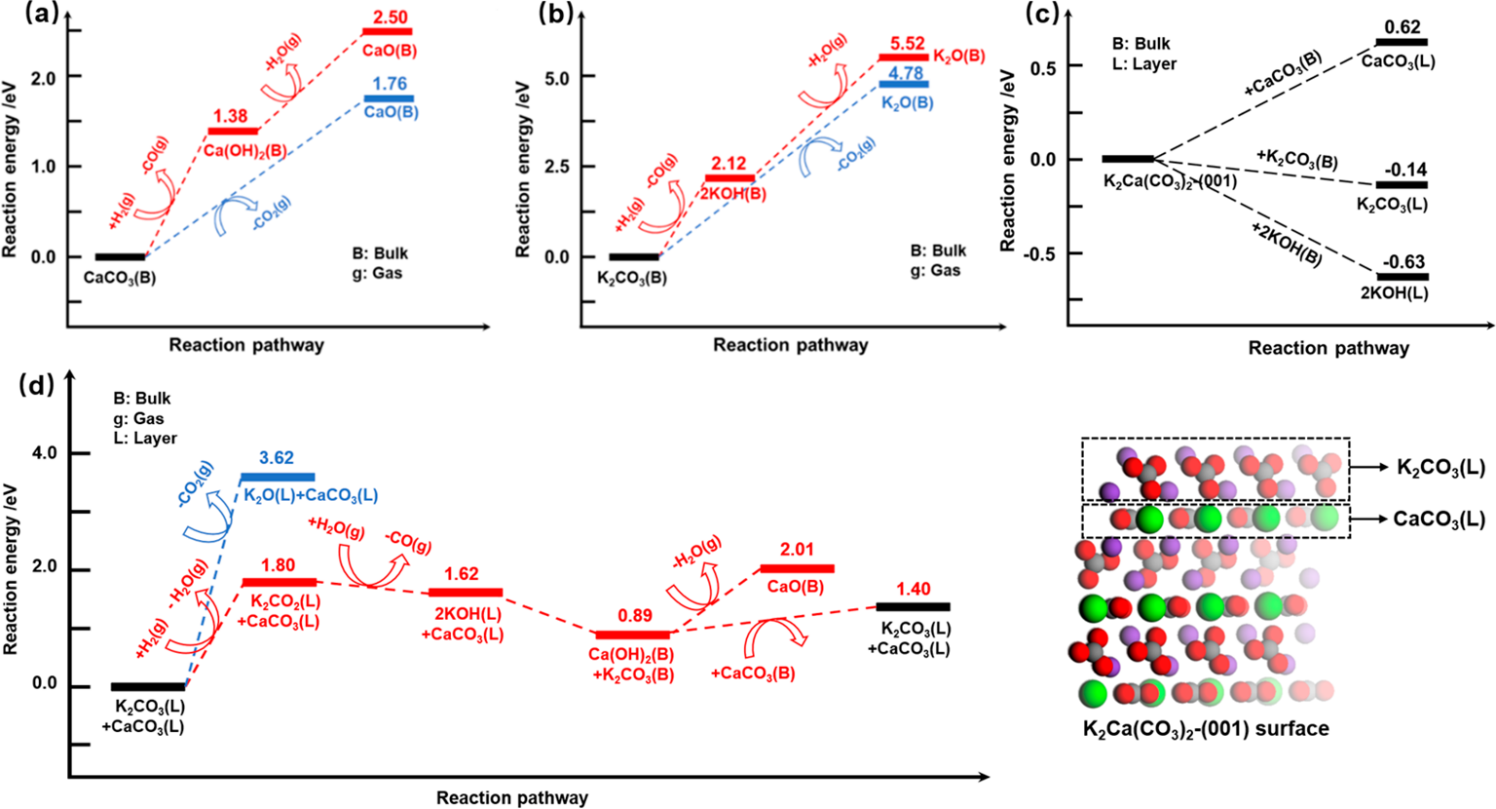
DFT calculations of CaCO_3_, K_2_CO_3_, and K_2_Ca(CO_3_)_2_ hydrogenation and decomposition. The calculated reaction energies of carbonates direct hydrogenation and decomposition over bulk CaCO_3_ (a) and bulk K_2_CO_3_ (b). Formation energies for layers of CaCO_3_, K_2_CO_3_, and 2KOH on the K_2_Ca(CO_3_)_2_-(001) surface from bulk CaCO_3_, K_2_CO_3_, and 2KOH, respectively (c). The calculated reaction energies of carbonates direct hydrogenation and decomposition over layers of CaCO_3_ and K_2_CO_3_ on the K_2_Ca(CO_3_)_2_-(001) surface (d). The C, O, K, and Ca were presented by the gray, red, purple, and green balls, respectively.

The consumption of carbonates in H_2_ atmosphere includes decomposition (reverse eq 3 or eq 8) and direct hydrogenation (eq 4 or eq 7) pathways, which could be illustrated by the isothermal thermogravimetric analysis under a N_2_ or H_2_ reducing atmosphere (Figure 2e). Consuming carbonate selectively through direct hydrogenation instead of decomposition is a widely overlooked but highly effective way to achieve a high CO_2_ conversion during the hydrogenation stage. It was found that the consumption rate of K_2_Ca(CO_3_)_2_ in 5% H_2_/Ar (eq 7, *k*_*2*_) was ∼6.1 times faster than its decomposition in N_2_ (eq 8, *k*_*1*_), while the consumption rate of CaCO_3_ was only 2.0 times faster in 5% H_2_/Ar than in N_2_. Compared to CaCO_3_, K_2_Ca(CO_3_)_2_ tends to be consumed via direct hydrogenation (eq 7) instead of decomposition (eq 8) in H_2_, indicating the high selectivity of direct hydrogenation of K_2_Ca(CO_3_)_2_. In short, as illustrated in Figure S18, K_2_Ca(CO_3_)_2_ is the key contributor to the 100% CO_2_ removal efficiency during carbonation due to the enhanced formation kinetics and also the enhanced CO_2_ conversion during hydrogenation via selectively converting carbonates through direct hydrogenation to produce CO (eq 7).
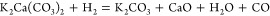
7
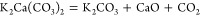
8To further study how K promotes the direct hydrogenation of carbonates to CO, DFT calculations were performed to compare the hydrogenation pathway with the decomposition pathway over CaCO_3_, K_2_CO_3_, and K_2_Ca(CO_3_)_2_, respectively. Since the reaction temperature was relatively high, we assumed that the calculated reaction energies provided a good estimation to compare the preferences of different reaction pathways. As shown in Figure 3a, the CaCO_3_ hydrogenation possessed a reaction energy of 1.38 eV to release CO and to form Ca(OH)_2_, which was actually observed in the in situ DRIFTs experiment (Figure 2g). The latter could decompose to (CaO + H_2_O) easily, as indicated by a further 1.12 eV decomposition energy. Even though the CaCO_3_ direct decomposition to (CaO + CO_2_) possessed a higher reaction energy of 1.76 eV, the difference was not high enough to avoid the CaCO_3_ direct decomposition in opposition to hydrogenation. These calculation results are consistent with the experimental observations that the hydrogenation rate of carbonates to release CO was higher than the rate of decomposition of CaCO_3_ (Figure 2e) which led to considerable CO_2_ release (Figure 2b), albeit with reasonable selectivity. Unlike bulk CaCO_3_, bulk K_2_CO_3_ was too stable to decompose, as indicated by a significantly higher calculated reaction energy of 4.78 eV (Figure 3b). In addition, the calculated reaction energy for K_2_CO_3_ hydrogenation to 2KOH was also quite high, at 2.12 eV, followed by an even higher dehydration reaction energy of 3.40 eV. Thus, neither decomposition nor hydrogenation of bulk K_2_CO_3_ was feasible, which was consistent with experimental observations performed by using bulk K_2_CO_3_ (Figure S19).

For the K-promoted CaO system, K_2_Ca(CO_3_)_2_ was found to form, consisting of interstitial layers (L) of CaCO_3_ and K_2_CO_3_ along the (001) direction (Figure S17). Our DFT calculations showed that the formation energy of K_2_CO_3_(L) and CaCO_3_(L) on the K_2_Ca(CO_3_)_2_-(001) surface from the bulk materials of K_2_CO_3_ and CaCO_3_ are −0.14 and 0.62 eV, respectively (Figure 3c). Thus, more stable K_2_CO_3_(L) was expected to be formed on the surface. Similar to bulk K_2_CO_3_, decomposition of K_2_CO_3_(L) was also unfavored, since the calculated reaction energy was 3.62 eV (Figure 3d). Meanwhile, the decomposition of nonpreferentially exposed CaCO_3_ (L) was found to be even more difficult (Figure S20). On the other hand, it was found that the hydrogenation of K_2_CO_3_(L) to the K_2_CO_2_(L) intermediate possessed a much lower reaction energy (1.80 eV, Figure 3d). Further hydrogenation resulted in 2KOH (L) with a formation energy of 1.62 eV with respect to K_2_CO_3_(L), although it was 0.24 eV higher than that of the CaCO_3_ hydrogenation to Ca(OH)_2_ (Figure 3a). These calculation results were consistent with the experimental observations that the K-promoted system had a high CO_2_ conversion to CO with ignorable CO_2_ emission (Figure 2a), although the hydrogenation rate was slower than that of bulk CaCO_3_. More importantly, although the KOH layer was found to be too stable to be decomposed, it could quickly react with CaCO_3_(L) with a reaction energy of −0.73 eV to form bulk Ca(OH)_2_ and K_2_CO_3_. The as-formed Ca(OH)_2_ (B) could decompose to generate CaO easily, while bulk K_2_CO_3_ could react with bulk CaCO_3_ to regenerate K_2_CO_3_(L) and CaCO_3_(L) with a reaction energy of 0.51 eV (Figure 3d). Therefore, DFT calculations suggested that the overall reaction pathway over the K-promoted CaO system involved hydrogenation of K_2_CO_3_ (L) to produce CO and an intermediate KOH layer on the surface of CaCO_3_ (L). Simultaneously, a new K_2_CO_3_ layer was expected to form on the surface of CaCO_3_ (L). The K_2_CO_3_ layer possesses superior selectivity to direct hydrogenation instead of decomposition and hence contributes to the excellent CO_2_ conversion in ICCU-RWGS using K-promoted CaO DFM.

## 3 Discussion

The ICCU process based on reverse water–gas shift (RWGS) reaction allows CO_2_ to be captured directly from flue gas and converted in situ into highly concentrated and valuable syngas. This provides a novel and promising engineering solution for achieving carbon neutrality. Although ICCU could achieve higher CO_2_ conversion rates compared to conventional RWGS, it is still restricted by the equilibrium of RWGS. A 20 mol % K-CaO DFM was produced here, exhibiting cyclically sustainable ∼100% CO_2_ removal efficiency from the flue gas and >95% CO_2_ conversion and ∼100% CO selectivity in hydrogenation at 650 °C over 50 reaction cycles, which outperforms the state-of-art counterparts. The performance evaluation, characterization, and simulation reveal that the direct gas–solid carbonates hydrogenation dominates in ICCU using K-CaO DFM, while the carbonates decomposition is highly suppressed due to the mainly formed K_2_Ca(CO_3_)_2_ with septal K_2_CO_3_ and CaCO_3_ layers. By improving the selectivity of carbonates direct hydrogenation out of decomposition, the ICCU using K-CaO DFM can bypass the gas–gas phase equilibrium restriction of RWGS, producing high purity syngas for the following applications. This work points out the immense potential of carbonate direct hydrogenation via a gas–solid phase CO_2_ conversion pathway in ICCU. Moreover, we show that unwanted side reactions such as the decomposition of carbonates can be hindered by introducing K to form intercalated CaCO_3_ bicarbonate layers. The process shown in this work represents a simple and effective strategy for future materials design in ICCU, which is significant for the deployment of ICCU technologies in real-life settings. More broadly, this work provides an alternative route for enhancing the catalytic efficiency of gas–gas phase reactions, which have significant implications for many other applications in sustainability beyond ICCU.

## 4 Methods

### 4.1 Material Preparation

The potassium-promoted CaO was prepared by wet impregnation with various molar ratios of potassium to calcium. Typically, as illustrated in Figure S2, *x* mol KNO_3_ (0 < *x* < 0.1; Sigma-Aldrich, >99%) was dissolved in 20 mL distilled water, followed by adding 0.1 – *x* mol CaCO_3_ (Sigma-Aldrich, >99%) into the aqueous solution. The mixture was stirred at room temperature for 1 h and vapored at 90 °C with continuous stirring and then dried at 110 °C overnight. The dried sample was ground and calcined at 800 °C for 5 h at a heating rate of 5 °C min^–1^. The obtained sample was named as *X* mol % K-CaO (*X* = 1000*x*, i.e., *x* = 0.01 for 10 mol % K-CaO).

### 4.2 Material Characterization

The K loading and Ca content were measured by inductively coupled plasma-optical emission spectroscopy (ICP-OES) using nitric acid digestion. The crystal structures of the materials were tested by powder X-ray diffraction (XRD) on a PANalytical empyrean series 2 diffractometer with a Cu Ka X-ray source. The attenuated total reflectance-Fourier transform infrared spectroscopy (ATR-FTIR) data were collected using Agilent Cary 630. The Raman spectra were characterized on the WITec Alpha 300R Confocal Raman Microscope equipped with a 532 nm diode laser (50 mW). The isothermal thermogravimetric analysis was carried out on a Hi-Res TGA 2950. High-res PXRD of the powder was carried out at beamline ID22 of the European Synchrotron Research Facility (ESRF) at Grenoble, France. The sample was packed into a 0.7 mm capillary that was sealed and mounted on a brass spinner. The sample was attached to a goniometer head and aligned to the beam spot. The diffraction pattern was collected at a wavelength of 0.3542 Å, while the capillary was in the rock mode. The textual information was collected by scanning electron microscopy (SEM) images coupled with an energy dispersive X-ray spectrometer (EDX) on FEI Quanta FEG. The surface area and pore structure of the materials were characterized by an ASAP 3000 analyzer, and the Brunauer–Emmett–Teller (BET) method was used to calculate the surface area. The in situ diffused reflectance infrared Fourier transform spectroscopy (in situ DRIFTs) experiments were carried out using an Agilent Cary 680 FTIR spectrometer with a liquid N_2_ cooled detector.

### 4.3 Performance Evaluation and Investigation

The ICCU was carried out in a quartz fixed bed reactor. The quartz reaction tube (12 mm OD; 10.5 mm ID and 650 mm length) was fixed in a tube furnace (Elite), and 0.5 g of sample was placed in the middle of the reaction tube and fixed with quartz wool. The mass of sample loading was calibrated by thermogravimetric analysis with 850 °C N_2_ calcination to eliminate the mass changes caused by the adsorption of substances in the air. The ICCU evaluation was isothermally carried out under 600, 650, and 700 °C, respectively. The real time gas concentration (CO_2_, CO, and CH_4_) during ICCU was monitored by an online gas analyzer (KANE AUTOplus 5–2) equipped with a nondispersive Infrared (NDIR) sensor. 15.2% CO_2_/N_2_ and 100% H_2_ were applied to simulate the flue gas and reducing agent for carbonation and hydrogenation, respectively. Typically, 100 mL min^–1^ (controlled by mass flow meter; OMEGA FMA-A2000) 15.3% CO_2_/N_2_ was introduced into the reaction tube for 30 min to demonstrate the CO_2_ capture procedure, then 100 mL min^–1^ N_2_ was introduced for 3 min to purge the residual gaseous CO_2_ followed by switching into 100 mL min^–1^ 100% H_2_ to achieve the CO generation and regeneration of sorbent. The CO_2_ conversion, CO selectivity and CO yield in hydrogenation were calculated by integrating the real-time data during hydrogenation, as shown in the following equations:
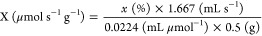
9
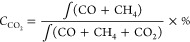
10
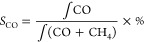
11
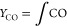
12X (μmol s^–1^ g^–1^; X = CO_2_, CO or CH_4_) represents the real-time flow rate of various fraction calculated from the percent fraction data. *C*_CO_2__ (%), *S*_CO_ (%), and *Y*_CO_ (mmol g^–1^) represent the CO_2_ conversion, CO selectivity, and CO yield.

### 4.4 Computational Details

All DFT calculations were performed with Vienna *ab initio* simulation package (VASP).^30,31^ The kinetic energy cutoff for the plane wave basis sets of the valence electrons was set to 400 eV. The core electrons were described by the projector augmented-wave (PAW) method.^32^ The surface Monkhorst–Pack meshes^33^ of 5 × 5 × 5 and 2 × 2 × 1 *k*-point sampling in the surface Brillouin zone were employed for the bulk and slab model, respectively. For bulk calculations, all atoms were relaxed, and the lattice constants were optimized. For surface slab modeling, the three bottom atomic layers were fixed while the other atomic layers were relaxed. After the convergence criteria for optimizations were met, the largest remaining force on each atom was less than 0.02 eV Å^–1^. For all calculations, the generalized gradient approximation (GGA) of the Perdew–Burke–Ernzerhof (PBE) functional^34^ was used. The contributions of dispersive interactions were accounted for by using the DFT+D3 method with Becke–Johnson (BJ) damping.^35,36^ The electronic energy was used for reaction energy calculations, which provided reaction energies of CaCO_3_ decomposition to produce CO_2_ (1.76 eV) and overall hydrogenation to produce CO and CaO (B) (2.50 eV) comparable to the experimental enthalpies presented in eq 3 (1.86 eV) and eq 4 (2.28 eV).

For calculating the formation energy of the K_2_CO_3_ layer and the KOH layer, the K_2_Ca(CO_3_)_2_-(001) surface with the CaCO_3_ layer ((001)-CaT) as the terminal layer was used. For calculating the formation energy of the CaCO_3_ layer, the K_2_Ca(CO_3_)_2_-(001) surface with the K_2_CO_3_ layer ((001)-KT) as the terminal was used. Using the K_2_CO_3_ layer as an example, the formation energy, Δ*E*_*f*_[K_2_CO_3_(L)], was calculated by
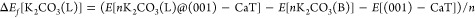
13where *n* represents the number of K_2_CO_3_ molecules loaded on the K_2_Ca(CO_3_)_2_-(001) surface, *E*[*n*K_2_CO_3_(B)] is *n* times of the total energy of bulk K_2_CO_3_, *E*[(001) – CaT] is the total energy of the K_2_Ca(CO_3_)_2_-(001) surface with the CaCO_3_ layer as terminal, and *E*[*n*K_2_CO_3_(B)@(001) – CaT] is the total energy of *n* K_2_CO_3_ loaded on the K_2_Ca(CO_3_)_2_-(001) surface with the CaCO_3_ layer as terminal.

## Supporting Information Available

Additional experimental details, materials characterizations, detailed data evaluation, and spectral assignment reference (PDF)
