# Supplementary material for: Potassium-Promoted Limestone for Preferential Direct Hydrogenation of Carbonates in Integrated CO2 Capture and Utilization
Source: JACS Au. 2023 Nov 9;4(1):72–9. doi: 10.1021/jacsau.3c00403 (PMC10806873; doi:10.1021/jacsau.3c00403)
Supplement: Supplementary file 1 — au3c00403_si_001.pdf [file au3c00403_si_001.pdf]

Supplementary Information

for

Potassium-promoted limestone for preferential direct hydrogenation of carbonates in integrated CO_2 capture and utilization

Shuzhuang Sun,[a,b‡] Zheng Chen,[c‡] Yikai Xu,[b,d] Yuanyuan Wang,[b] Yingrui Zhang,[b] Catherine Dejoie,[e] Shaojun Xu,[f,g*] Xin Xu,[c*] and Chunfei Wu[b*]

[a] *School of Chemical Engineering, Zhengzhou University, Zhengzhou, 450001, China*

[b] *School of Chemistry and Chemical Engineering, Queen's University Belfast, Belfast, BT7 1NN, UK*

[c] *Department of Chemistry, Fudan University, Shanghai, 200433, China*

[d] *Key Laboratory for Advanced Materials and Feringa Nobel Prize Scientist Joint Research Center, Frontiers Science Center for Materiobiology and Dynamic Chemistry, School of Chemistry and Molecular Engineering, East China University of Science and Technology, 130 Meilong Road, Shanghai 200237, China*

[e] *European Synchrotron Radiation Facility, Grenoble, 38043, France*

[f] *Department of Chemical Engineering, University of Manchester, Manchester M13 9PL, UK*

[g] *UK Catalysis Hub, Research Complex at Harwell, Didcot, OX11 0FA, UK*

Corresponding authors: Shaojun Xu: xus25@cardiff.ac.uk; Xin Xu: xxchem@fudan.edu.cn; Chunfei Wu: c.wu@qub.ac.uk

‡These authors contributed equally to this work.

Contents

Supporting figures and tables (Page 3-26)
References (Page 26-27)

Supporting figures and tables

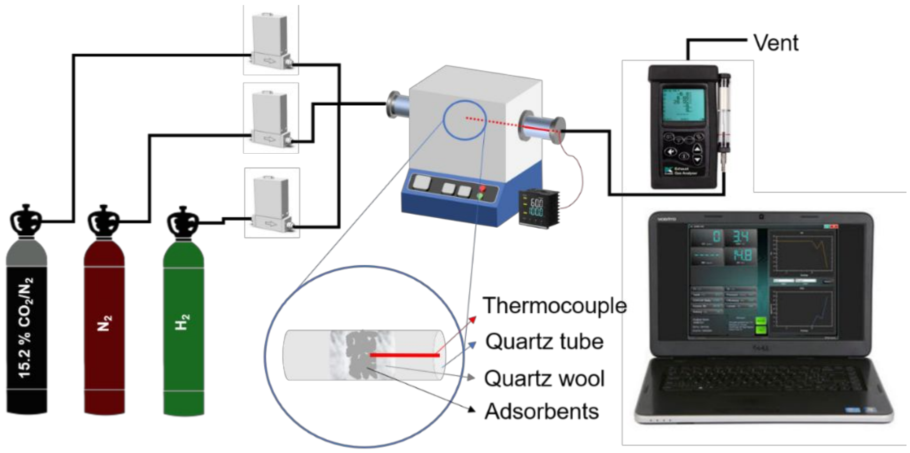

Figure S1. Schematic diagram of the integrated CO_2 capture and conversion system.

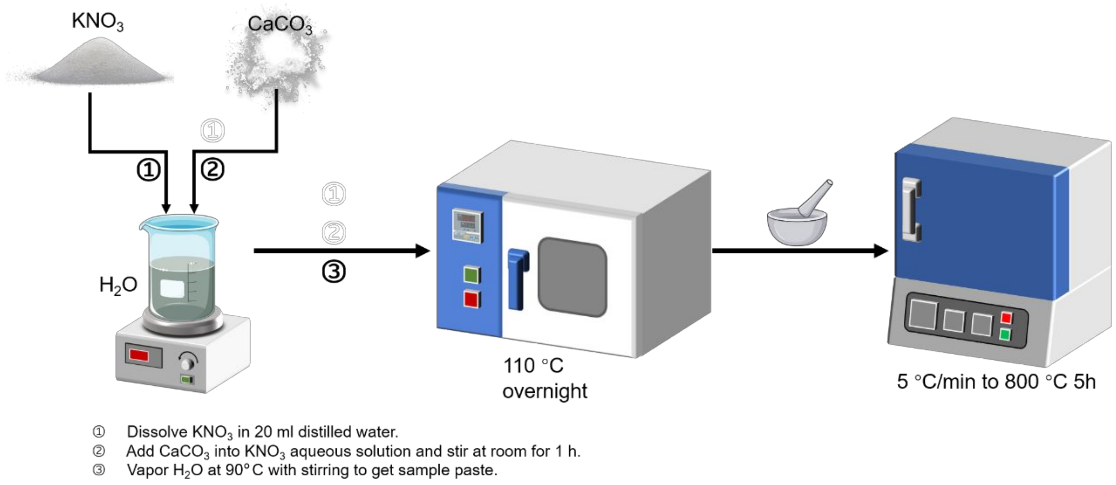

Figure S2. Schematic diagram of the preparation of K-promoted CaO materials.

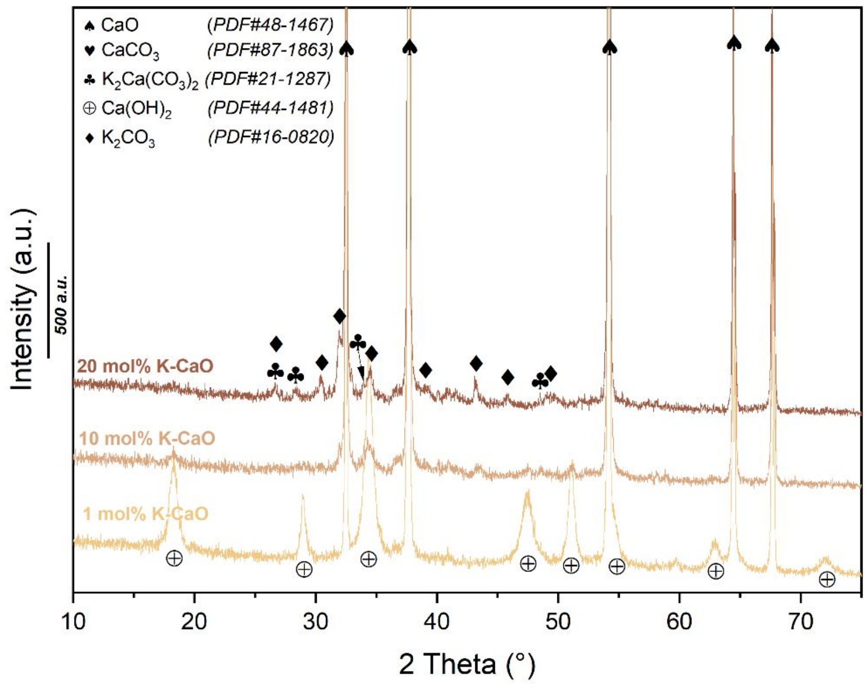

Figure S3. XRD patterns of original and K-promoted CaO with different K loadings.

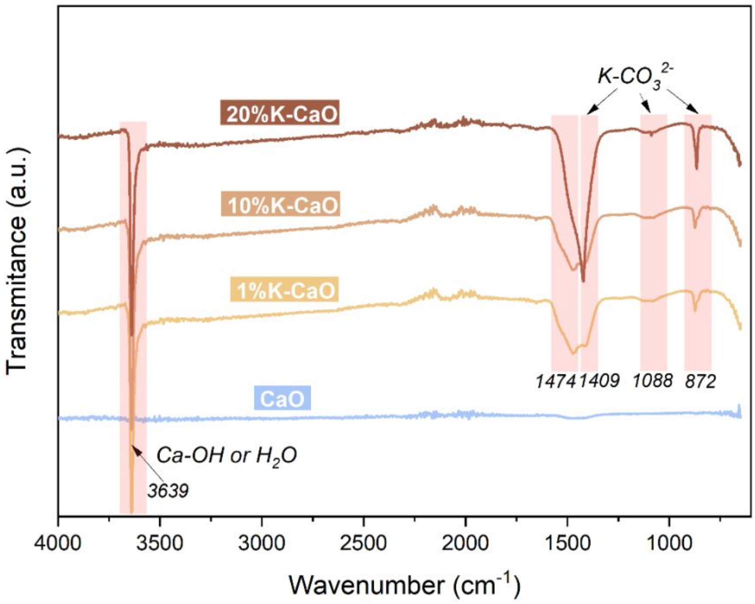

Figure S4. ATR-FTIR patterns of original and K-promoted CaO with different K loadings.

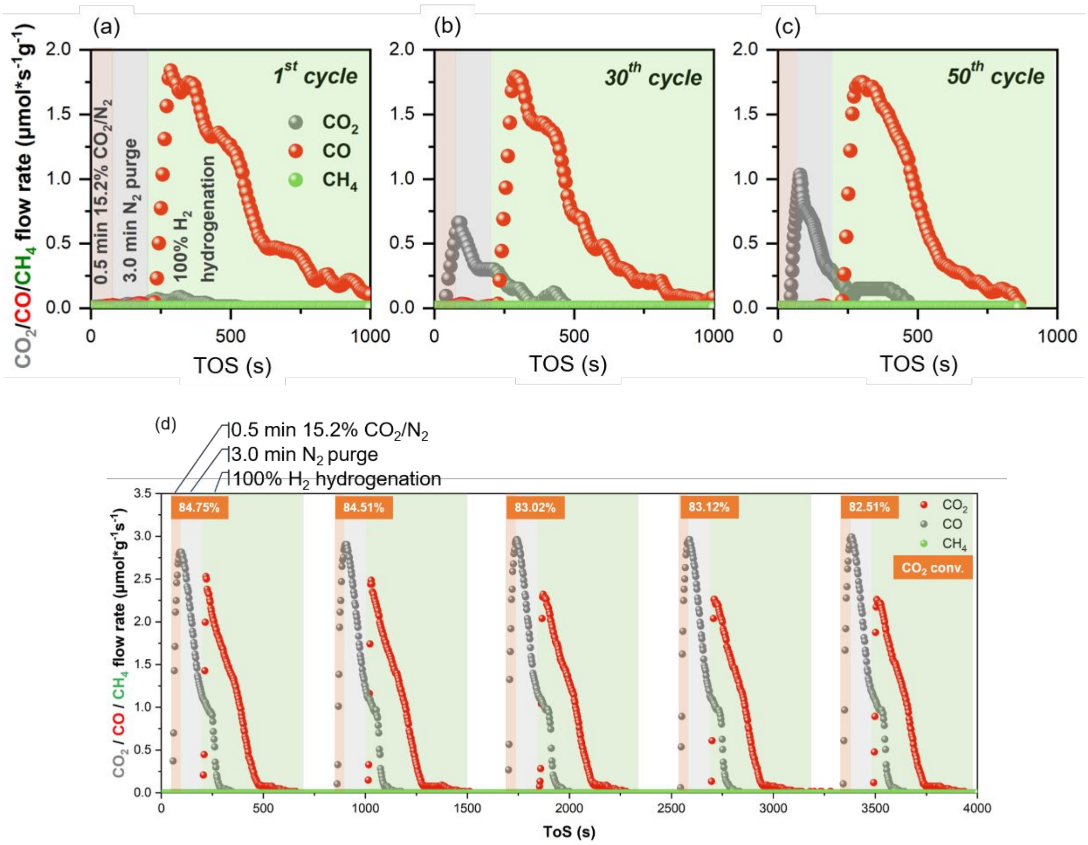

Figure S5. Real time cyclic ICCU performance of 20 mol% K-CaO at 650 °C at (a) 1st cycle; (b) 30th cycle and (c) 50th cycle. And real time cyclic (5 cycles) ICCU performance of CaO at 650 °C. (E_{CO2} represents CO_2 removal efficiency calculated by the integration of CO_2 flow rate and benchmark data of CO_2 baseline; ICCU procedure: Carbonation: 15.2% CO_2/N_2 for 0.5 min; Purge: N_2 for 3.0 min; Hydrogenation: 100% H_2 to the end)

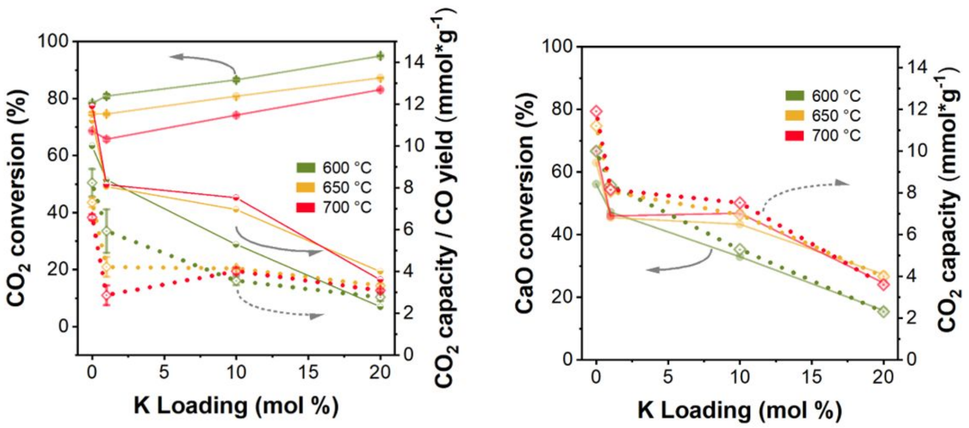

Figure S6. CO_2 conversion, CO_2 capacity and CO yield in the ICCU over K-CaO in relation to K loading and temperature (Carbonation: 15.2% CO_2/N_2 for 30 min; Purge: N_2 for 3.0 min; Hydrogenation: 100% H_2 to the end).

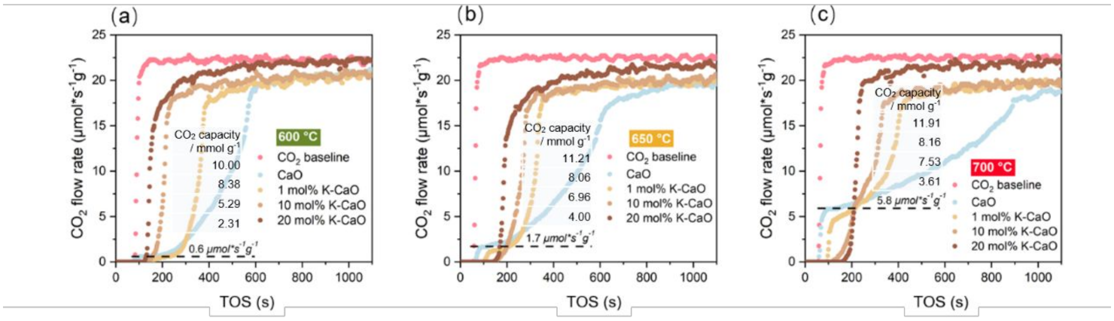

Figure S7. Real time CO_2 adsorption performance of CaO and K-promoted CaO at (a) 600 °C, (b) 650 °C and (c) 700 °C. (ICCU procedure: Carbonation: 15.2% CO_2/N_2 for 30 min; Purge: N_2 for 3.0 min; Hydrogenation: 100% H_2 to the end)

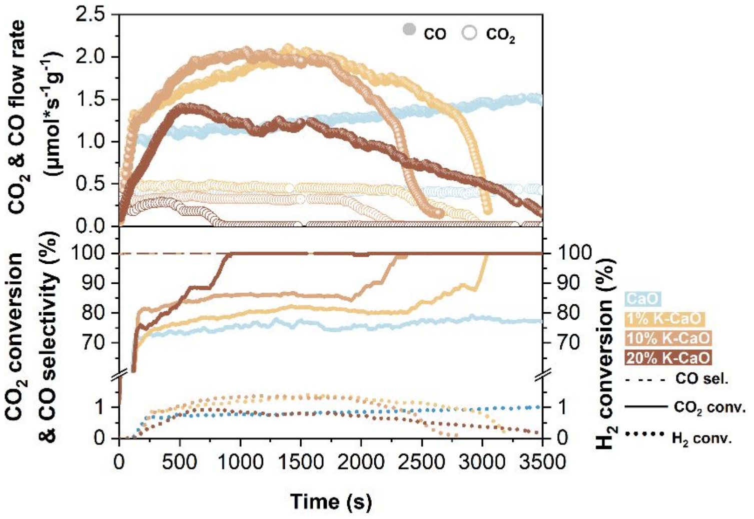

Figure S8. Real time hydrogenation performance in ICCU (CO_2 and CO generation flow rate, CO_2 conversion and CO selectivity) over K-promoted CaO at 600 °C. (ICCU procedure: Carbonation: 15.2% CO_2/N_2 for 30 min; Purge: N_2 for 3.0 min; Hydrogenation: 100% H_2 to end)

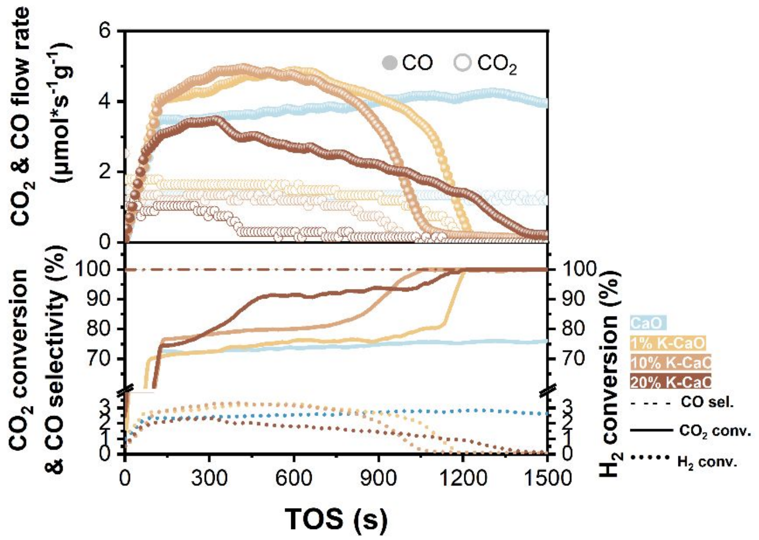

Figure S9. Real time hydrogenation performance in ICCU (CO_2 and CO generation flow rate, CO_2 conversion and CO selectivity) over K-promoted CaO at 650 °C. (ICCU procedure: Carbonation: 15.2% CO_2/N_2 for 30 min; Purge: N_2 for 3.0 min; Hydrogenation: 100% H_2 to end)

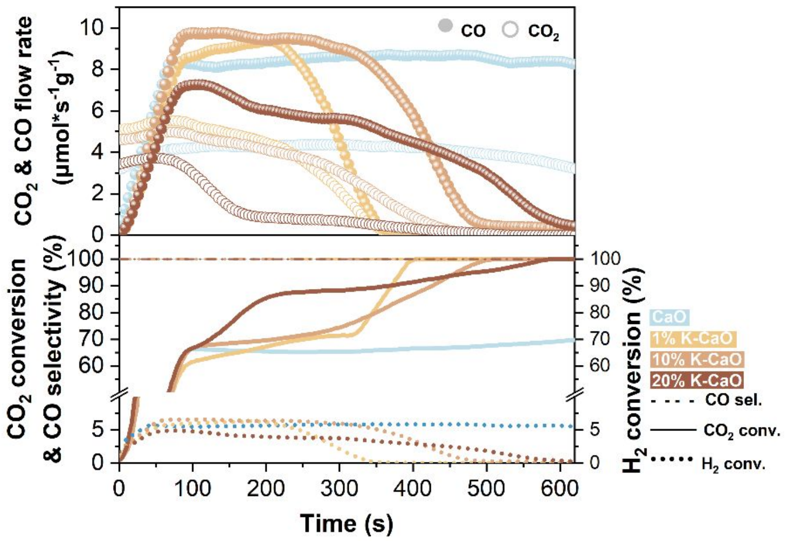

Figure S10. Real time hydrogenation performance in ICCU (CO_2 and CO generation flow rate, CO_2 conversion and CO selectivity) over K-promoted CaO at 700 °C. (ICCU procedure: Carbonation: 15.2% CO_2/N_2 for 30 min; Purge: N_2 for 3.0 min; Hydrogenation: 100% H_2 to end)

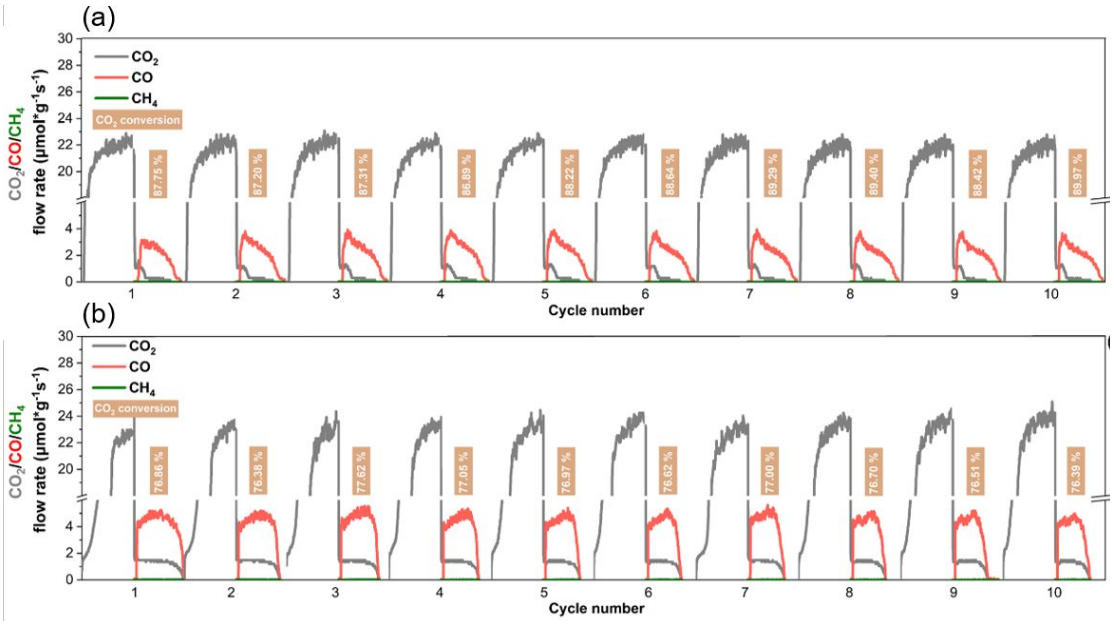

Figure S11. Cyclic performance of ICCU (10 cycles) over (a)20 mol% K-CaO and (b)CaO at 650 °C. (ICCU procedure: Carbonation: 15.2% CO_2/N_2 for 30 min; Purge: N_2 for 3.0 min; Hydrogenation: 100% H_2 to end)

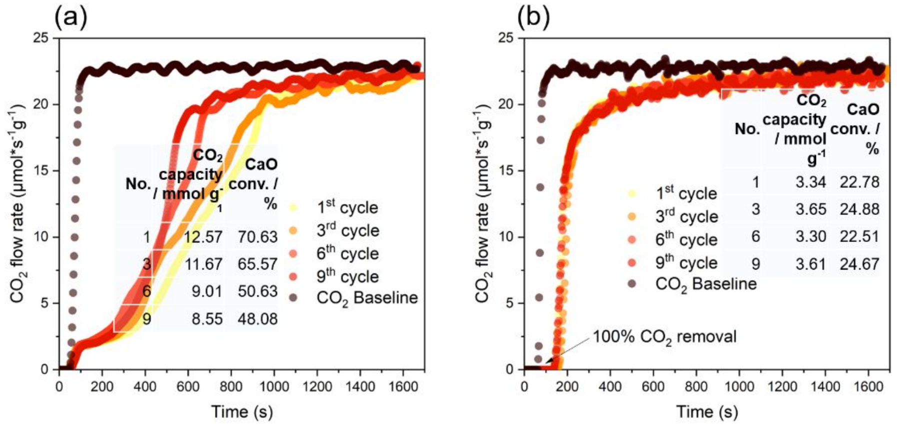

Figure S12. Real time CO_2 adsorption performance of (a) CaO and (b) 20mol% K-CaO in 10 ICCU cycles at 650 °C.

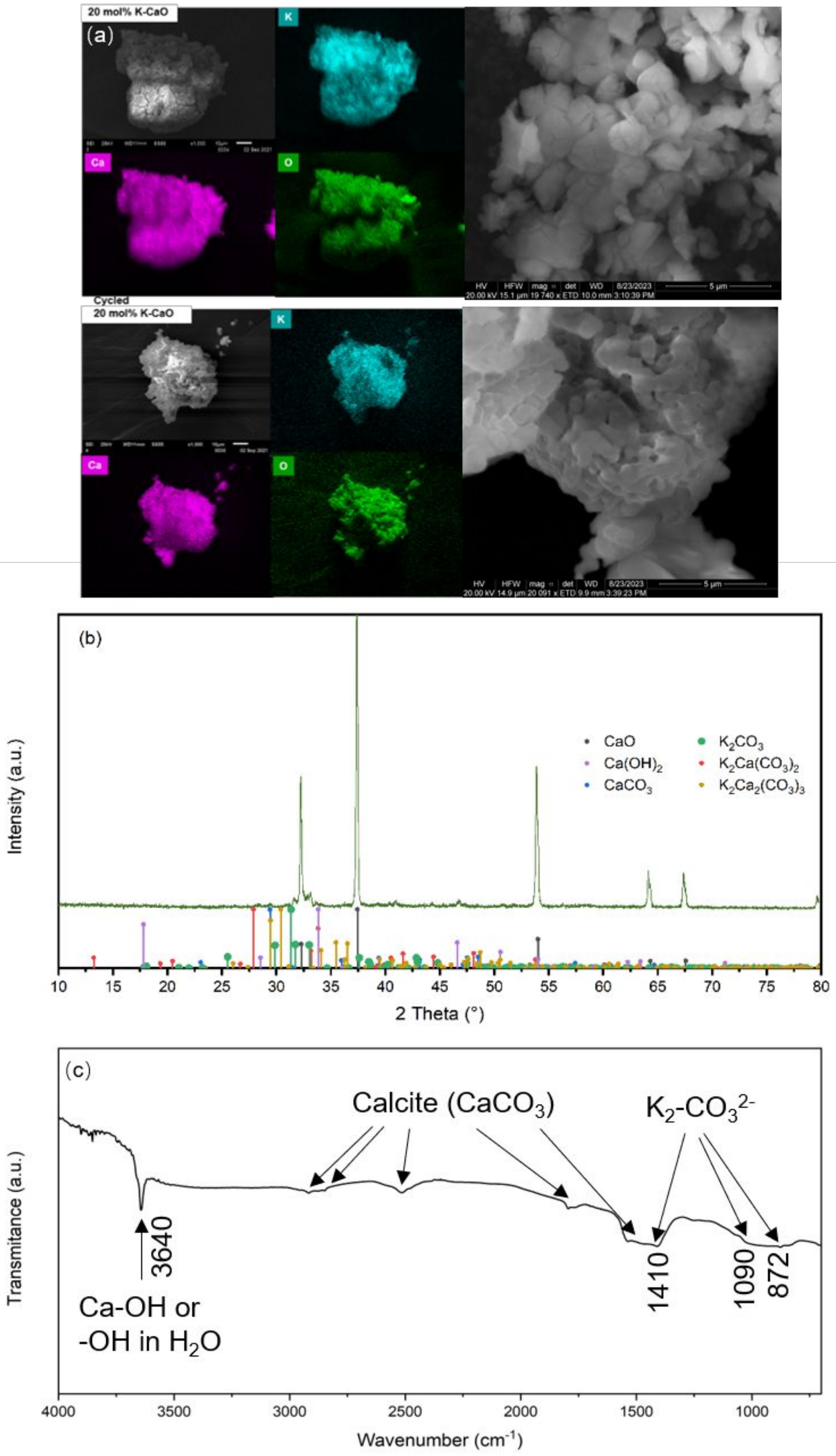

Figure S13. (a) SEM and element mapping images of original and cycled 20 mol% K-CaO; (b) XRD and (c) FTIR of cycled 20 mol% K-CaO.

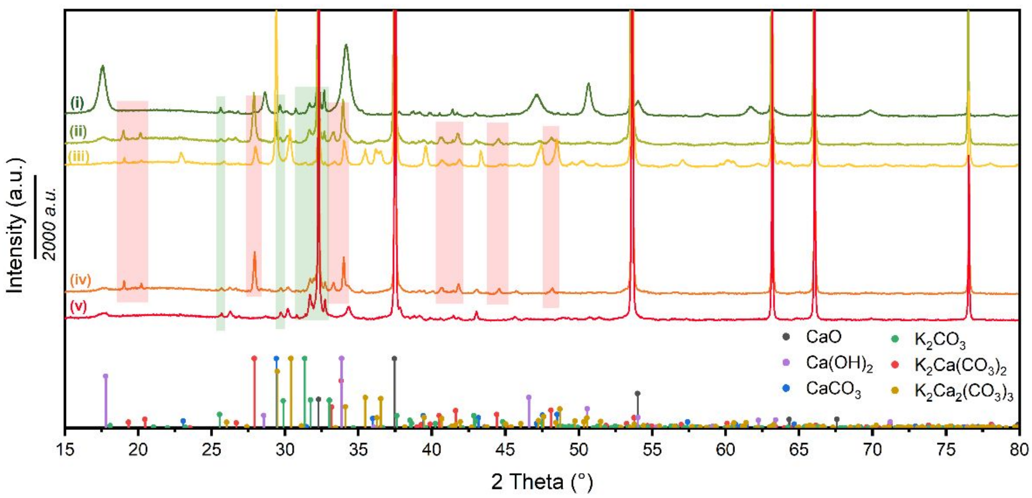

Figure S14. Ex-situ SRXRD of 20 mol% K-CaO in ICCU at 650 °C. (Carbonation: 15.2% CO_2/N_2 for 30 min; Purge: N_2 for 3.0 min; Hydrogenation: 100% H_2 to end; i: original material; ii: 0.5 min carbonation; iii: 30 min carbonation; iv: 10 min hydrogenation and v: end of hydrogenation)

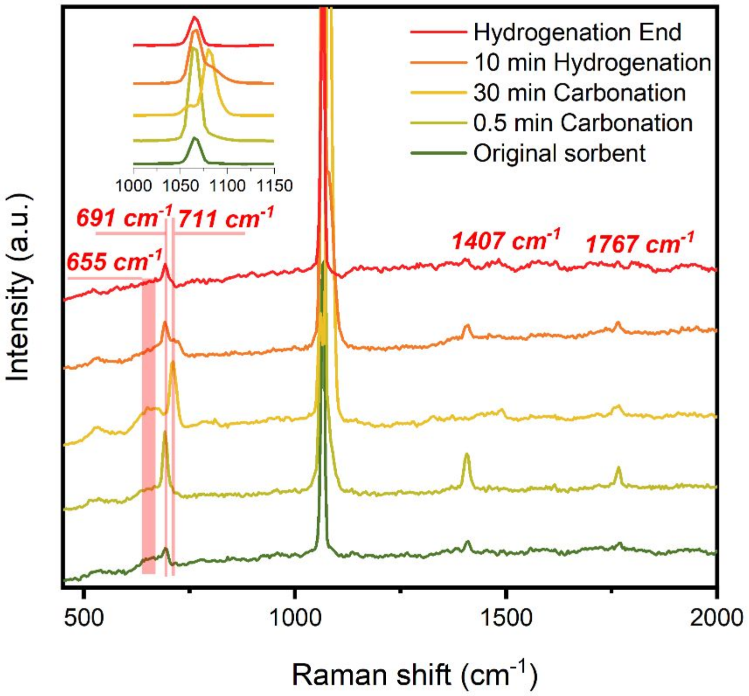

Figure S15. Ex-situ Raman spectrum of 20 mol% K-CaO in ICCU at 650 °C. (Carbonation: 15.2% CO_2/N_2 for 30 min; Purge: N_2 for 3.0 min; Hydrogenation: 100% H_2 to end)

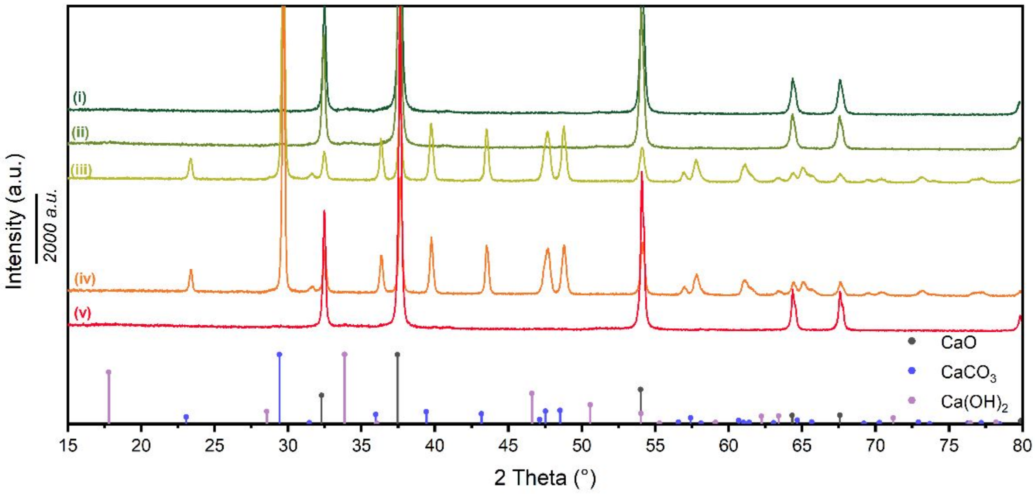

Figure S16. Ex-situ XRD of CaO in ICCU at 650 °C. (Carbonation: 15.2% CO_2/N_2 for 30 min; Purge: N_2 for 3.0 min; Hydrogenation: 100% H_2 to end; i: original material; ii: 0.5 min carbonation; iii: 30 min carbonation; iv: 10 min hydrogenation and v: end of hydrogenation)

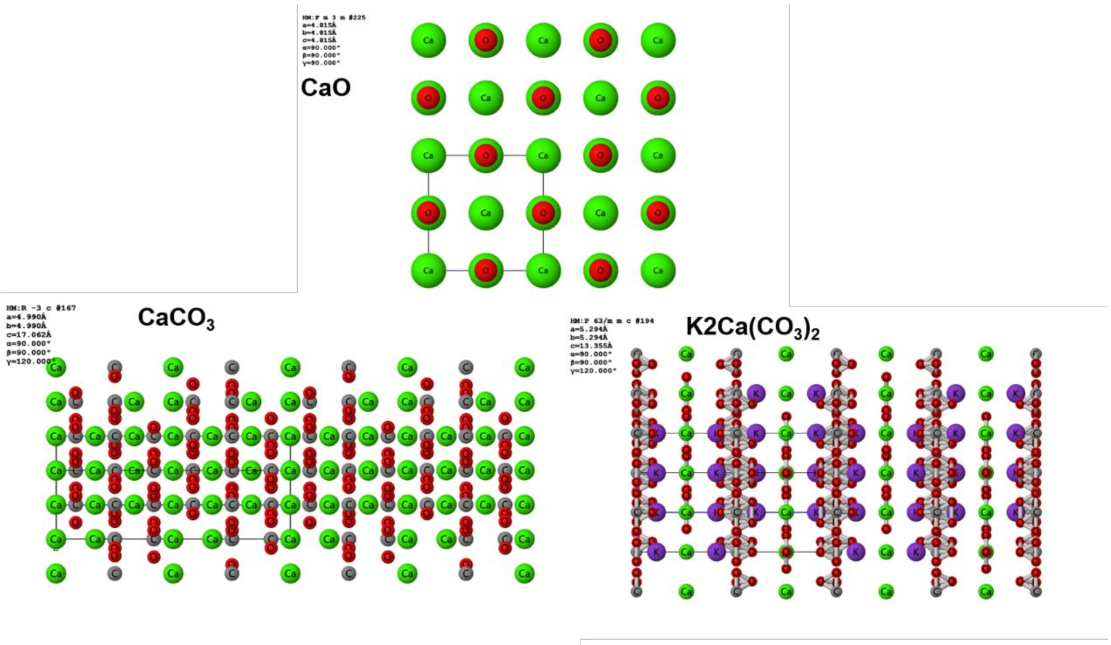

Figure S17. Crystal structure of CaO, $CaCO_3$ and $K_2Ca(CO_3)_2$.

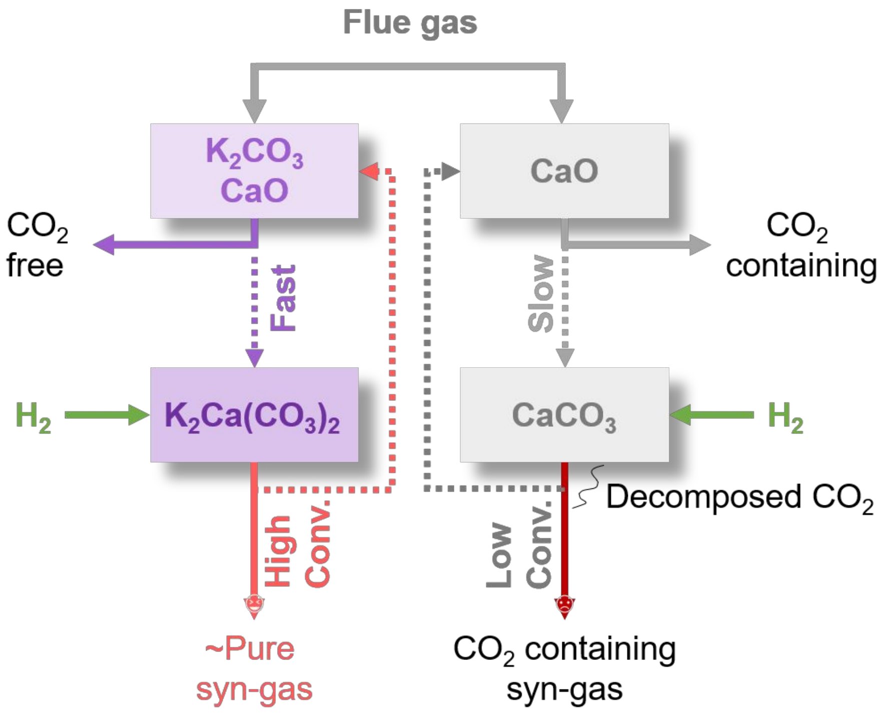

Figure S18. ICCU schematic comparison of CaO with and without K promotion.

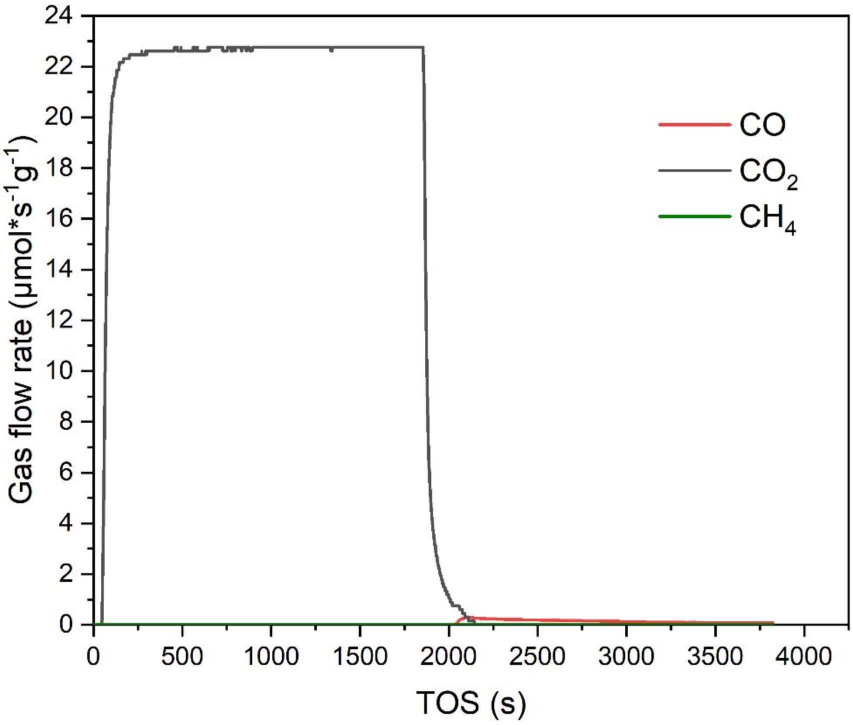

Figure S19. Real time ICCU performance using K_2CO_3 reagent at 650 °C (Carbonation: 15.2% CO_2/N_2 for 30 min; Purge: N_2 for 3.0 min; Hydrogenation: 100% H_2 to the end). Note: The weak CO generation might be attributed to the surface adsorbed CO_2 hydrogenation or the slow surface reaction of K_2CO_3.

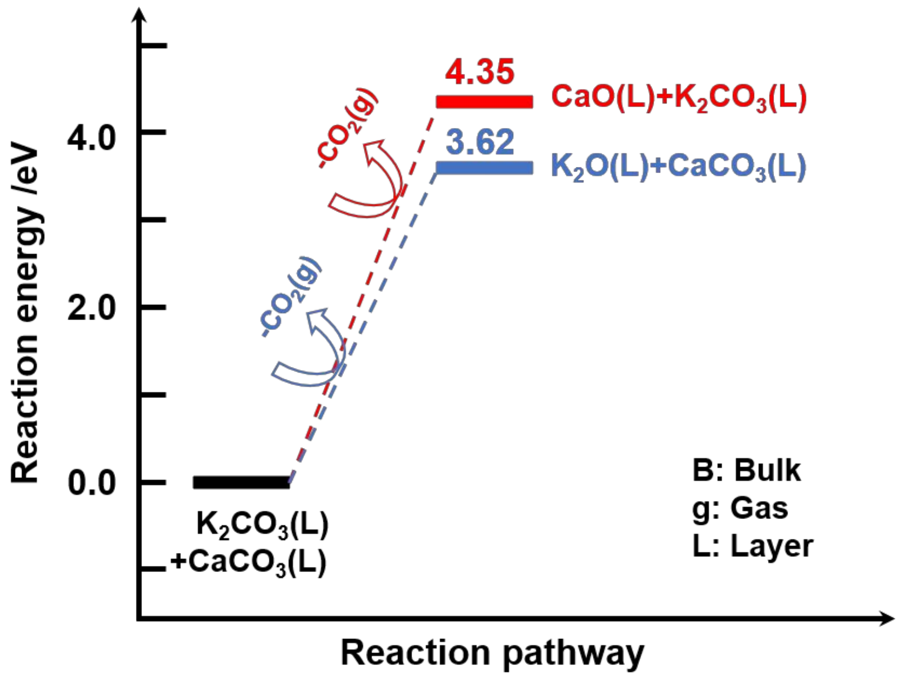

Figure S20. The calculated reaction energies of the decomposition of $CaCO_3$ and K_2CO_3 layers on $K_2Ca(CO_3)_2$.

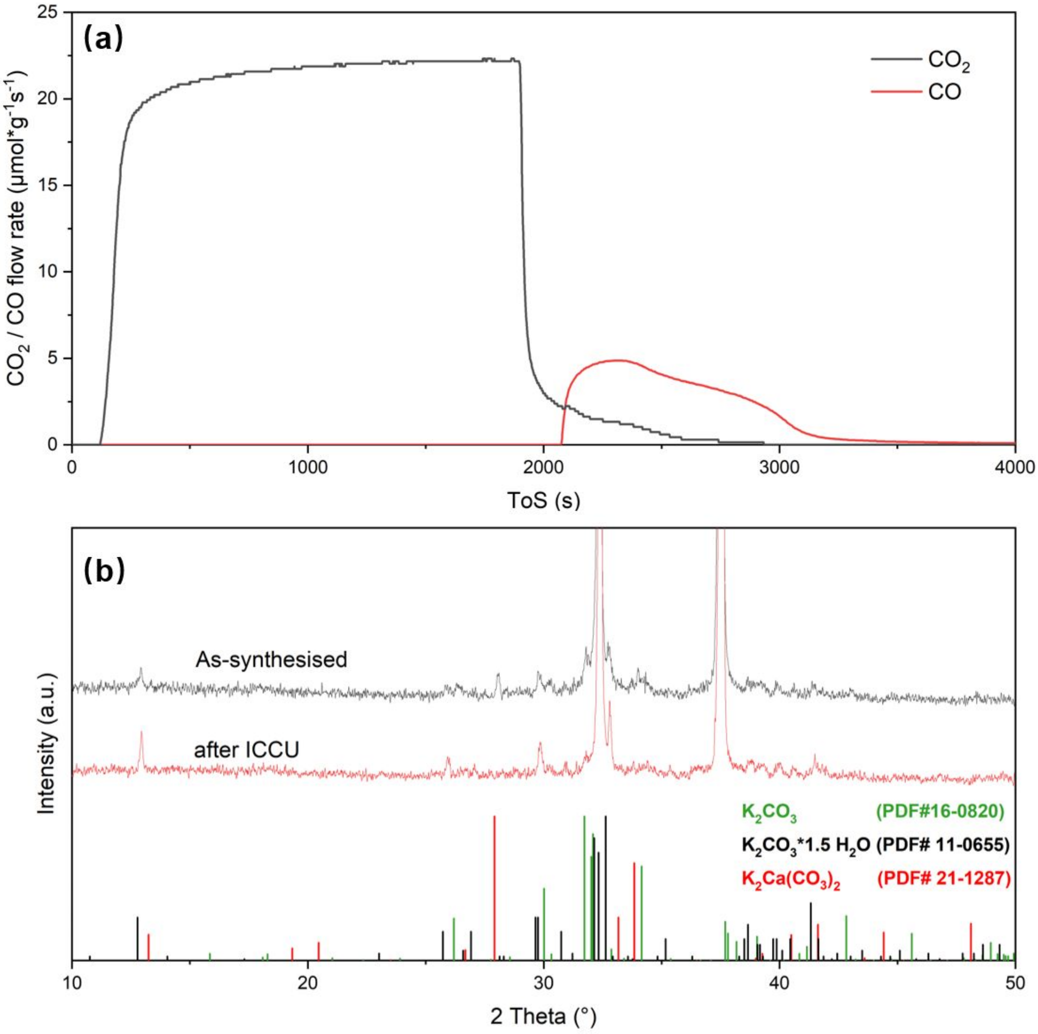

Figure S21. ICCU performance of 20% K-CaO using KOH as a precursor (a) and the XRD patterns of the as-synthesized and ICCU-reacted 20% K-CaO (b).

Table S1 Literature comparison of CaO-based materials for ICCU-RWGS

No.	Materials	CO_2 adsorption step	Hydrogenation step	CO_2 escape flow in capture μmol/g*s	CO_2 conversion	CO selectivity	Ref.
1	20 mol% K-CaO	22.6 μmol/g*s CO_2 (balanced in 126.2 μmol/g*s N_2), 650 °C	148.8 μmol/g*s H_2, 650 °C	0	>95%	>99.9%	This work
2	$Ca_1Ni_{0.1}Ce_{0.033}$	44.7μmol/g*s CO_2 (balanced in 252.9 μmol/g*s N_2), 650 °C	14.9 μmol/g*s H_2 (balanced in 282.8 μmol/g*s N_2), 650 °C	>0.83	51.8%	n.a.	[8]
3	Ni_1Fe_9-CaO	24.8 μmol/g*s CO_2 (balanced in 223.3 μmol/g*s N_2), 650 °C	248.1 μmol/g*s H_2, 650 °C	>3.23	82.7%	99.8%	[9]
4	Ni/CS-P30-C	26.8 μmol/g*s CO_2 (balanced in 241.1 μmol/g*s N_2), 650 °C	13.4 μmol/g*s H_2 (balanced in 254.5 μmol/g*s N_2), 650 °C	n.a.	38.6%	n.a.	[10]
5	$Fe_5Co_5Mg_{10}CaO$	14.9 μmol/g*s CO_2 (balanced in 133.9 μmol/g*s N_2), 650 °C	89.3 μmol/g*s H_2, 650 °C	>2	90%	n.a.	[11]

Table S2. K loading and BET surface area of CaO and K-promoted CaO

Sorbent Name	K loading (by mol) K/(K+Ca)[a]	S_{BET} (m^2*g^{-1})
CaO	n.a.	7.08
1 mol% K-CaO	0.3%	2.84
10 mol% K-CaO	7.9%	4.17
20 mol% K-CaO	14.8%	0.36
50 cycled 20 mol% K-CaO	12.7%	4.21

[a] measured by ICP-OES.

Table S3. Raman bands references for ex-situ Raman of 20 mol% K-CaO using 532 cm^{-1} laser.

Ref	Phase	v_1	v_2	v_3	v_4	v_1+v_4	Eg	O-H
12	K_2CO_3	~1062		~1400	~690	~1752		
13	$CaCO_3$	~1070	868	~1390-1460	~698-723	~1751		
14	$K_2Ca(CO_3)_2$	~1063-1077		~1400-1520	~704-719	~1740		
14	$K_2Ca_2(CO_3)_3$	~1077	~820-875	~1402-1487	~705-711	~1753-1765		
15	$Ca(OH)_2$						~684	~3620

v_1—mode of symmetric stretching vibrations; v_2—mode of out-of-plane symmetric vibrations; v_3—mode of in-plane bending vibrations; v_4—mode of asymmetric stretching vibrations; v_1+ v_4—combination modes.

Note: CaO was reported not to possess any first-order and second-order Raman active modes due to the weakness and the overlap with the bonds of $Ca(OH)_2$[16], which are located at ~ 650 and 3619 cm^{-1}.

Reference

1 Kresse, G. & Furthmüller, J. Efficiency of ab-initio total energy calculations for metals and semiconductors using a plane-wave basis set. *Computational materials science* **6**, 15-50 (1996). https://doi.org:10.1016/0927-0256(96)00008-0

2 Kresse, G. & Furthmüller, J. Efficient iterative schemes for ab initio total-energy calculations using a plane-wave basis set. *Physical review B* **54**, 11169 (1996). https://doi.org:10.1103/PhysRevB.54.11169

3 Blöchl, P. E. Projector augmented-wave method. *Physical review B* **50**, 17953 (1994). https://doi.org:10.1103/PhysRevB.50.17953

4 Monkhorst, H. J. & Pack, J. D. Special points for Brillouin-zone integrations. *Physical review B* **13**, 5188 (1976). https://doi.org:10.1103/PhysRevB.13.5188

5 Perdew, J. P., Burke, K. & Ernzerhof, M. Generalized gradient approximation made simple. *Physical review letters* **77**, 3865 (1996). https://doi.org:10.1103/PhysRevLett.77.3865

6 Grimme, S., Antony, J., Ehrlich, S. & Krieg, H. A consistent and accurate ab initio parametrization of density functional dispersion correction (DFT-D) for the 94 elements H-Pu. *The Journal of chemical physics* **132**, 154104 (2010). https://doi.org:10.1063/1.3382344

7 Grimme, S., Ehrlich, S. & Goerigk, L. Effect of the damping function in dispersion corrected density functional theory. *Journal of computational chemistry* **32**, 1456-1465 (2011). https://doi.org:10.1002/jcc.21759

8 Sun, H. *et al.* Dual functional catalytic materials of Ni over Ce-modified CaO sorbents for integrated CO_2 capture and conversion. *Applied Catalysis B: Environmental* **244**, 63-75 (2019). https://doi.org:10.1016/j.apcatb.2018.11.040

9 Sun, S., He, S. & Wu, C. Ni promoted Fe-CaO dual functional materials for calcium chemical dual looping. *Chem. Eng. J.* **441**, 135752 (2022). https://doi.org:10.1016/j.cej.2022.135752

10 Wang, G. *et al.* Ni-CaO dual function materials prepared by different synthetic modes for integrated CO_2 capture and conversion. *Chem. Eng. J.* **428**, 132110 (2022). https://doi.org:10.1016/j.cej.2021.132110

11 Shao, B. *et al.* Heterojunction-redox catalysts of $Fe_xCo_yMg_{10}CaO$ for high-temperature CO_2 capture and in situ conversion in the context of green manufacturing. *Energy Environ. Sci.* **14**, 2291-2301 (2021). https://doi.org:10.1039/D0EE03320K

12 Maciel, A., Ryan, J. & Walker, P. Structural phase transitions in K_2CO_3 (raman scattering study). *Journal of Physics C: Solid State Physics* **14**, 1611 (1981).

13 Tlili, M. *et al.* Characterization of $CaCO_3$ hydrates by micro‐Raman spectroscopy. *Journal of Raman spectroscopy* **33**, 10-16 (2002). https://doi.org:10.1002/jrs.806

14 Arefiev, A., Podborodnikov, I., Shatskiy, A. & Litasov, K. Synthesis and

Raman Spectra of K–Ca Double Carbonates: $K_2Ca(CO_3)_2$ Bütschliite, Fairchildite, and $K_2Ca_2(CO_3)_3$ at 1 Atm. *Geochemistry International* **57**, 981-987 (2019).
15 Padanyi, Z. The Raman spectrum of $Ca(OH)_2$. *Solid State Communications* **8**, 541-543 (1970).
16 Schmid, T. & Dariz, P. Shedding light onto the spectra of lime: Raman and luminescence bands of CaO, $Ca(OH)_2$ and $CaCO_3$. *Journal of Raman Spectroscopy* **46**, 141-146 (2015).
